# Hydrophilic Auristatin Glycoside Payload Enables Improved Antibody-Drug Conjugate Efficacy and Biocompatibility

**DOI:** 10.3390/antib7020015

**Published:** 2018-03-22

**Authors:** Tero Satomaa, Henna Pynnönen, Anja Vilkman, Titta Kotiranta, Virve Pitkänen, Annamari Heiskanen, Bram Herpers, Leo S. Price, Jari Helin, Juhani Saarinen

**Affiliations:** 1 Glykos Finland Ltd., FI-00790 Helsinki, Finland; henna.pynnonen@glykos.fi (H.P.); anja.vilkman@glykos.fi (A.V.); titta.kotiranta@glykos.fi (T.K.); virve.pitkanen@glykos.fi (V.P.); annamari.heiskanen@glykos.fi (A.H.); jari.helin@glykos.fi (J.H.); 2 OcellO B.V., 2333 CH Leiden, The Netherlands; bram.herpers@ocello.nl (B.H.); leo.price@ocello.nl (L.S.P.)

**Keywords:** glycoside, auristatin, MMAE, MMAU, ADC, hydrophilicity, therapeutic window

## Abstract

Antibody-drug conjugates (ADCs) offer a combination of antibody therapy and specific delivery of potent small-molecule payloads to target cells. The properties of the ADC molecule are determined by the balance of its components. The efficacy of the payload component increases with higher drug-to-antibody ratio (DAR), while homogeneous DAR = 8 ADCs are easily prepared by conjugation to the four accessible antibody hinge cystines. However, use of hydrophobic payloads has permitted only DAR = 2–4, due to poor pharmacokinetics and aggregation problems. Here, we describe generation and characterization of homogeneous DAR = 8 ADCs carrying a novel auristatin β-D-glucuronide, MMAU. The glycoside payload contributed to overall hydrophilicity of the ADC reducing aggregation. Compared to standard DAR = 2–4 ADCs, cytotoxicity of the homogeneous DAR = 8 ADCs was improved to low-picomolar IC_50_ values against cancer cells in vitro. Bystander efficacy was restored after ADC internalization and subsequent cleavage of the glycoside, although unconjugated MMAU was relatively non-toxic to cells. DAR = 8 MMAU ADCs were effective against target antigen-expressing xenograft tumors. The ADCs were also studied in 3D in vitro patient-derived xenograft (PDX) assays where they outperformed clinically used ADC. In conclusion, increased hydrophilicity of the payload contributed to the ADC’s hydrophilicity, stability and safety to non-target cells, while significantly improving cytotoxicity and enabling bystander efficacy.

## 1. Introduction

There are currently four clinically approved antibody-drug conjugates (ADCs) in the market, directed against both hematologic malignancies and solid tumors. ADCs utilize highly potent cytotoxic payloads, originally discovered as having anti-tumor activity, but then proven to be too toxic for human use in the form of small-molecule drugs. However, utilizing tumor-specific antibodies as targeting vehicles and development of sophisticated linker technologies has since enabled clinical use of these potent anticancer compounds [1]. That being said, the therapeutic window of ADCs is still quite narrow and many clinical programs have suffered from unwanted toxicity and insufficient efficacy against tumors. Therefore, development of more safe and efficacious ADC technology with a wider therapeutic window remains a viable field of study. It has been established that ADCs with higher drug-to-antibody ratio (DAR) have greater in vitro potency than the current clinically approved ADCs with DAR of about four, and the higher DAR ADCs are especially effective against cells with low copy numbers of the target antigen [2]. However, DAR = 8 monomethylauristatin E (MMAE) ADCs are inferior to both DAR = 2 and DAR = 4 ADCs in vivo due to their poor in vivo pharmacokinetics and systemic exposure [2,3]. Further studies have convincingly demonstrated that this effect was due to the hydrophobic character of the current ADC payloads and that by masking the hydrophobic payloads by hydrophilic linker moieties DAR = 8 ADCs with improved in vivo biodistribution and efficacy can be achieved [4].

**Figure 1:**
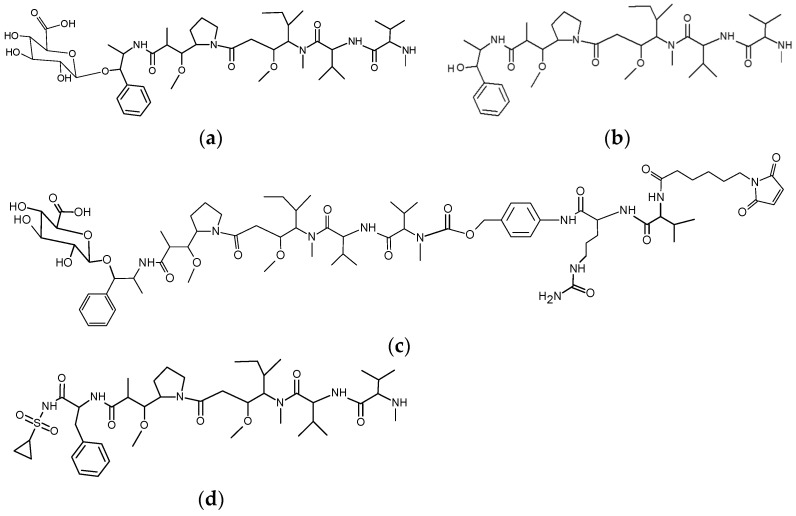
Structures of the payloads and linkers in the present study. (**a**) MMAU, β-D-glucuronyl-monomethylauristatin E; (**b**) MMAE, monomethylauristatin E; (**c**) MC-Val-Cit-PABC-MMAU, ε-maleimidocaproyl-L-valine-L-citrulline-paraaminobenzyloxycarbonyl-MMAU; (**d**) Duostatin 3. Hydrophilic and lysosomally cleavable glycoside payload MMAU allows for generation of homogeneous drug-to-antibody ratio (DAR) = 8 antibody-drug conjugates (ADCs) with high efficacy, safety to non-target cells and utilization of the bystander effect.

We took another line of study and reasoned that the same effect could be achieved by transient hydrophilic modification of the payload itself. We synthesized hydrophilic glycoside-modified versions of common small-molecule hydrophobic ADC payloads to make them more hydrophilic. In the present study we describe novel glycosides of the tubulin polymerization inhibitor MMAE that has a hydroxyl functional group useful for substitution with O-glycosidic linkage (Figure 1). The glycosides were designed to be stable in the bloodstream and extracellular space while being readily hydrolysable by lysosomal glycosidases so that the original payload molecule would be specifically liberated in active form inside target cells. In the course of the study we sought to evaluate if DAR = 8 ADCs would be feasible with this approach, without compromising the performance of the resulting auristatin ADCs.

## 2. Materials and Methods

### 2.1. Preparation of Drug-Linker Compounds

A total of 1.9 mg (2.2 µmol) β-D-glucuronyl-monomethylauristatin E (MMAU; [5]) was dissolved in dimethylformamide (DMF, 200 µL) and combined with 1.9 molar excess of ε-maleimidocaproyl-l-valine-l-citrulline-paraaminobenzyloxycarbonyl-paranitrophenyl (MC-Val-Cit-PABC-pNP; Levena Biopharma, San Diego, CA, USA), 2 µL 0.5 M hydroxybenzotriazole (HOBt, 1 µmol) and 3 µL diisopropylethylamine (17 µmol). The reaction was stirred overnight at room temperature. The resulting drug-linker MC-Val-Cit-PABC-MMAU was purified by HPLC with Gemini 5 µm NX-C18 AXIA reversed-phase column (21.1 × 250 mm, 110 Å; Phenomenex, Værløse, Denmark) eluted with acetonitrile gradient in aqueous ammonium acetate. The eluted fractions were analyzed by MALDI-TOF mass spectrometry with a Bruker Ultraflex III TOF/TOF mass spectrometer (Bruker Daltonics Inc., Bremen, Germany) using 2,5-dihydroxybenzoic acid matrix (DHB) and the mass spectrum of the purified compound is presented in Supplementary Figure S1 showing the expected mass (*m*/*z* 1515.0, [M + Na]^+^). In later work, MC-Val-Cit-PABC-MMAU was supplied by Levena Biopharma and characterized by a combination of analytical chromatography, mass spectrometry and weighing of the dried compound. The corresponding non-glycosidic drug-linker MC-Val-Cit-PABC-MMAE was obtained directly from Levena Biopharma.

### 2.2. Preparation and Characterization of Antibody-Drug Conjugates

Interchain disulphide bridges of trastuzumab (Herceptin^®^, Roche Pharma AG, Grenzach-Wyhlen, Germany) were reduced with tris(2-carboxyethyl)phosphine (TCEP): 0.1 mM antibody was incubated with 1 mM TCEP and 1 mM triamine pentaacetic acid (DTPA) in phosphate-buffered saline (PBS) for 1.5 h at +37 °C. TCEP was removed by repeated additions of 1 mM DTPA-5% mannitol-0.1% Tween-PBS and centrifugation through Amicon Ultracel 30 K centrifugal filter (Merck Millipore Ltd., Tullagreen, Ireland). ADCs were synthesized by incubating 0.1 mM antibody with 50× molar excess of either MC-Val-Cit-PABC-MMAU or MC-Val-Cit-PABC-MMAE drug-linker compound in 20% 1,2-propanediol-0.8 mM DTPA-4% mannitol-0.08% Tween-PBS for 1 h at room temperature. Prior to conjugation, the drug-linkers were dissolved in dimethylsulfoxide (DMSO) to reach 10% DMSO in the final reaction solution. Non-conjugated drug-linkers were removed by repeated additions of formulation buffer (5% mannitol-0.1% Tween-PBS) and centrifugation through the Amicon filter.

For analysis of the drug-to-antibody ratio (DAR), 30 µg sample of each ADC was digested with 34 U FabRICATOR enzyme (Genovis, Lund, Sweden) at +37 °C for 2 h, purified with Poros R1 tips (ThermoFisher Scientific, Vantaa, Finland) and analyzed by MALDI-TOF mass spectrometry using sinapinic acid matrix. MMAU-PABC-Cit-Val-MC-ADC light chains (LC) were observed at *m*/*z* 24,926 (LC + MMAU), Fab heavy chain fragments (Fab-HC) were observed at *m/z* 29854 (Fab-HC + 3 MMAU) and the Fc heavy chain fragments (Fc) were observed at *m/z* 25,227, 25,389 and 25,551 for differentially galactosylated fragments G0F-Fc, G1F-Fc and G2F-Fc, respectively (without C-terminal lysine). Thus the analysis demonstrated that all the eight solvent-accessible cysteine residues had been reduced and conjugated with drug-linker yielding an ADC with drug-to-antibody ratio 8 (DAR = 8). The MMAE-PABC-Cit-Val-MC-ADC, as well as ADCs based on cetuximab (Erbitux^®^, Merck KGaA, Darmstadt, Germany) were similarly analyzed and found to have DAR = 8. Anti-HER2 duostatin-3 ADCs were prepared similarly as described [6].

### 2.3. Hydrophobic Interaction Chromatography

To study their relative hydrophilicity, the naked antibody and ADCs were subjected to hydrophobic interaction chromatography (HIC-HPLC) using TSKgel Butyl-NPR column (0.46 × 3.5 cm; Tosoh Bioscience, South San Francisco, CA, USA). The column was equilibrated with 1.5 M ammonium sulphate, 25 mM potassium phosphate, pH 7.0, and run with a linear gradient over 15 min to 25 mM potassium phosphate, pH 7.0, containing 25% isopropanol at ambient temperature. Absorbance at 280 nm was recorded. The naked antibody eluted with least retention, followed by the ADCs in the order of increasing hydrophobicity. Standard compound mixture was generated by mixing trastuzumab and ADCs with different drug-to-antibody ratios; the ADCs were generated from reduced trastuzumab by adding varying amounts of the MC-Val-Cit-PABC-MMAE drug-linker compound until DAR = 8 was reached. The mixture was then applied to the HIC-HPLC column in one run.

### 2.4. Aggregation Assay

DAR = 8 ADCs were placed to heat stress conditions at +40 °C at a concentration of 4.6 mg/mL. The aggregation status was analyzed by size-exclusion chromatography (SEC-HPLC) on a 1.0 × 30 cm column of Superdex 200, in an isocratic run with 0.2 M potassium phosphate, 0.25 M potassium chloride, pH 7.0. Control ADCs were stored at +4 °C. Aggregated high-molecular weight components (HMWCs) eluted before the elution position of the non-aggregated control ADC and control naked antibody, and the relative molar proportions of the components were calculated based on integrated absorbance at 280 nm.

### 2.5. Cytotoxicity Assays

In vitro cytotoxicity of the free payloads and ADCs was assayed similarly as described [6,7]. IC_50_ values were determined using curve fitting by nonlinear regression as the concentration of the drug that causes 50% inhibition of cell viability compared to maximum inhibition. The 3D in vitro patient-derived xenograft (PDX) assays were performed with the GXA3067 gastric cancer model essentially as described [8]. PDX tumor was obtained from Charles River Labs, Freiburg, Germany. T-DM1 (Kadcyla^®^) was from Roche Pharma AG (Grenzach-Wyhlen, Germany).

### 2.6. In Vivo Xenograft Experiments

The animal experiments were performed at the Turku Center for Disease Modeling, Turku, Finland, with approval of the appropriate ethics committee according to license ESAVI/1993/04.10.03/2011, PH336A. EGFR-expressing human HSC-2 head-and-neck squamous cell carcinoma cells (ATCC) were injected subcutaneously into adult female Envigo HSD:Athymic nude Foxn1nu mice. Tumors were allowed to grow to approximately 100 mm^3^ (about 6 mm diameter) before the first treatment. ADC treatment was given intravenously four times at seven day intervals. Tumor volume was followed regularly. End-point of the study was when the tumors reach the maximum allowed diameter (17 mm) or at least 90 days had passed from the start of the experiment.

## 3. Results

### 3.1. Glycoside-Modified Auristatin ADCs

**Figure 2:**
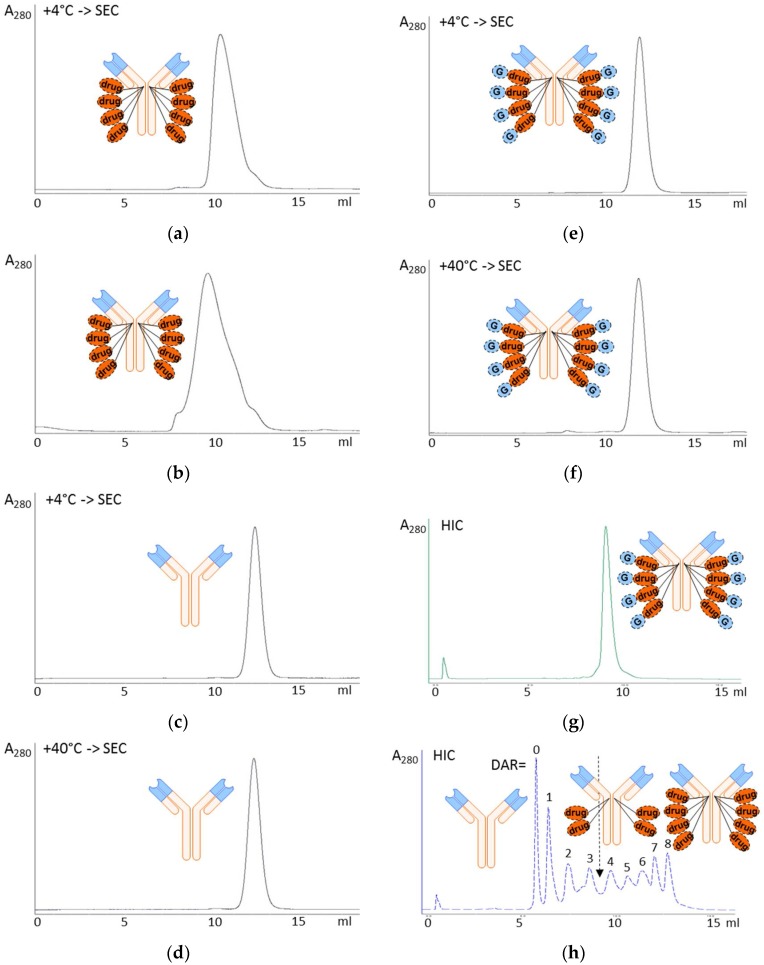
Chromatographic evaluation of ADC aggregation and hydrophilicity. (**a**–**f**) Aggregation was analyzed by size-exclusion chromatography (SEC) after two days’ storage at +4 °C (normal storage temperature) or +40 °C (temperature stress) in formulation buffer. (**a**,**b**) DAR = 8 trastuzumab MMAE ADC was moderately aggregated into HMWCs already at +4 °C in two days (**a**) and mostly (>95%) aggregated at +40 °C eluting as a broad peak between 7–12 mL (**b**). (**c**,**d**) Trastuzumab, the naked antibody control, was not aggregated at either +4 °C (**c**) or +40 °C (**d**) during storage and eluted at 12.1 mL. (**e**,**f**) DAR = 8 MMAU ADC was not aggregated at +4 °C eluting at 11.9 mL (**e**) and only 2% aggregated into high-molecular weight components (HMWCs) eluting at 7–8 mL at +40 °C (**f**). (**g**,**h**) Hydrophobic interaction chromatography (HIC) was performed for evaluating the relative hydrophilicity of DAR = 8 trastuzumab-MMAU ADC (**g**); and a standard mixture of the naked antibody trastuzumab as well as trastuzumab-MMAE ADCs with DAR = 1–8 constructed from reduced trastuzumab using standard MC-Val-Cit-PABC linker (**h**). The method separated the molecules in the order of relative hydrophobicity, with the most hydrophilic component (naked antibody) eluting first and the most hydrophobic component (DAR = 8 ADC) eluting last. DAR = 8 MMAU ADC eluted at position corresponding to DAR = 3–4 MMAE ADC. Detection was by absorbance at 280 nm. drug, MMAE; G, β-D-glucuronic acid glycoside.

Homogeneous DAR = 8 ADCs were successfully prepared from MMAU (Figure 1a), the β-D-glucuronic acid glycoside of MMAE (Figure 1b). For the present study we used standard valine-citrulline peptide linker (Figure 1c) with maleimide coupling to reduced antibody hinge region cysteines. We had hypothesized that more hydrophilic payloads would give improved resistance to aggregation during storage compared to regular MMAE. The aggregation propensity of glycoside ADCs was initially evaluated by size-exclusion chromatography after elevated temperature stress at +40 °C, showing that DAR = 8 MMAU ADC was resistant to aggregation (only 2% aggregated into high-molecular weight components, HMWCs) in conditions where DAR = 8 MMAE ADC was mostly aggregated (>95% HMWCs) and the naked antibody showed no aggregation (Figure 2a–f). The auristatin glycoside MMAU was clearly more hydrophilic than the parent compound MMAE, as evidenced by capillary electrophoresis experiments which will be published elsewhere [9]. In the present study, the relative hydrophilicity of the whole ADCs was evaluated by hydrophobic interaction chromatography (Figure 2g,h), showing that hydrophilicity of DAR = 8 MMAU ADC was between DAR = 3 MMAE ADC and DAR = 4 MMAE ADC. Since the approved MMAE ADC, Adcetris^®^, has an average DAR = 3.6 [10], we concluded that suitable hydrophilicity had been achieved.

### 3.2. In Vitro Efficacy Evaluation

**Figure 3:**
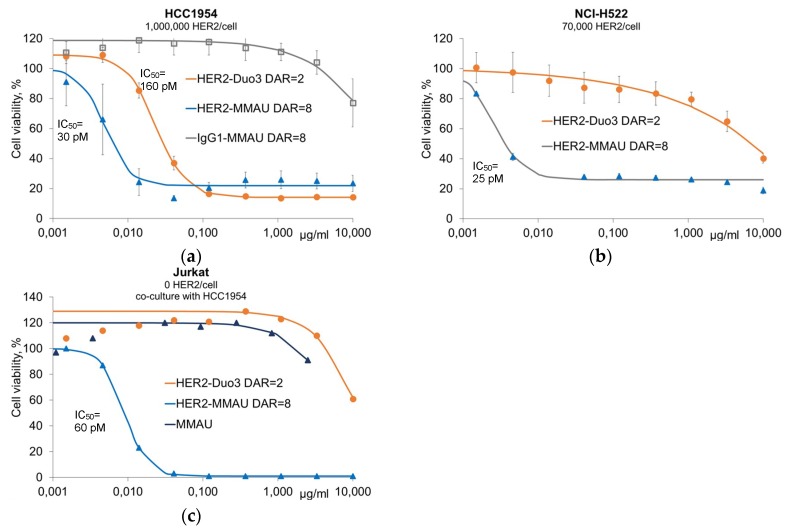
In vitro cytotoxicity of anti-HER2 ADCs against HER2-expressing cancer cell lines. (**a**) For high copy number HER2-expressing cells, both anti-HER2 DAR = 8 MMAU ADC and DAR = 2 duostatin-3 ADC showed high cytotoxic activity, while both anti-HER2 naked antibody and an isotype IgG control antibody-MMAU conjugate showed only no or low cytotoxicity, respectively. All experiments were performed in triplicate except for anti-HER2 naked antibody; (**b**) For low copy number HER2-expressing cells, the anti-HER2 DAR = 8 MMAU ADC had superior activity compared with DAR = 2 duostatin-3 ADC. Both experiments were performed in triplicate; (**c**) To demonstrate bystander kill activity of MMAU ADCs, non-HER2-expressing Jurkat cells were co-cultured with HCC1954 cells that express HER2, and viability of Jurkat cells was measured after incubation with fluorescence-assisted cell sorting (FACS)-based assay. The anti-HER2 DAR = 8 MMAU ADC showed bystander kill activity, while neither the anti-HER2 DAR = 2 duostatin-3 ADC nor the free MMAU payload had cytotoxic activity towards Jurkat cells. The results are from a single representative experiment. IC_50_ values are marked in the figure panels and they were determined using curve fitting by nonlinear regression as the concentration of the drug that causes 50% inhibition of cell viability compared to maximum inhibition. Error bars show the standard error of the mean, where applicable.

Anti-HER2 ADCs were evaluated in vitro against HER2-expressing and non-expressing cancer cell lines: high-copy number HCC1954 cells and low-copy number NCI-H522 cells as well as Jurkat cells that do not express HER2 (Figure 3). These cell lines express the GUSB gene encoding the human lysosomal β-glucuronidase enzyme [11]. With the high-copy number cells, both anti-HER2 DAR = 8 MMAU ADC (IC_50_ = 30 pM) and DAR = 2 duostatin-3 ADC (IC_50_ = 160 pM) showed good cytotoxic activity (Figure 3a), while with the low-copy number HER2-expressing cells the DAR = 8 MMAU ADC had superior activity with similar IC_50_ value (25 pM) as with the high-copy number cells (Figure 3b). This may be interpreted so that when the internalization rate of the ADC is low, high DAR becomes crucial, as previously established by others [12]. Since MMAU was designed to be cleaved by lysosomal β-glucuronidase enzyme to the hydrophobic drug MMAE, we were interested in whether MMAU ADCs would show bystander kill activity. To demonstrate the bystander efficacy, non-HER2-expressing Jurkat cells were co-cultured with HCC1954 cells that express HER2 and incubated with either ADCs or free MMAU. The anti-HER2 DAR = 8 MMAU ADC showed bystander kill activity (IC_50_ = 60 pM), while neither the anti-HER2 DAR = 2 duostatin-3 ADC [6] nor the free MMAU payload had cytotoxic activity towards Jurkat cells, as expected (Figure 3c).

### 3.3. In Vivo Experiments

**Figure 4:**
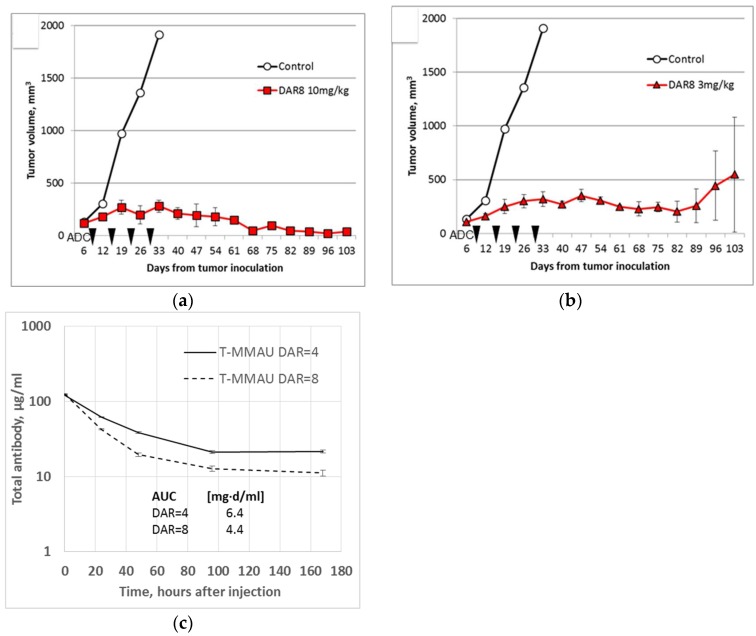
In vivo experiments. (**a**,**b**) EGFR^+^ HSC-2 tumor xenografts in mice were effectively treated with anti-EGFR DAR = 8 MMAU ADCs based on cetuximab. Mice were inoculated with tumor cells and treatment was given intravenously either with (**a**) 10 mg/kg or (**b**) 3 mg/kg ADC once weekly for four weeks (arrows). With the larger dose (**a**) the tumors shrunk permanently (follow-up of 100 days) and with the lower dose (**b**) the growth of the tumors was arrested in all but one mice (recurrence after 80 days). There were three mice in each treatment group. Error bars show the standard error of the mean. (**c**) Trastuzumab-MMAU ADCs (T-MMAU) were given to nude mice as single 10 mg/kg i.v. injection. DAR = 8 MMAU ADC showed 31% smaller area under curve (AUC) than DAR = 4 MMAU ADC. There were three mice in each treatment group. Error bars show the standard deviation.

To demonstrate that the MMAU ADCs have efficacy against solid tumors in vivo, subcutaneous EGFR+ HSC-2 tumor xenografts were established in nude mice. The HSC-2 cell line has been shown to express the GUSB gene encoding the human lysosomal β-glucuronidase enzyme [11], and the ADCs showed expected cytotoxicity in vitro (data not shown). After the tumors had reached 100 mm^3^ size, the mice were given once weekly intravenous (i.v.) treatment with DAR = 8 MMAU ADCs for four weeks. Treatment with both 10 mg/kg doses (Figure 4a) or 3 mg/kg doses (Figure 4b) were effective compared to the control treatment with only buffer, directly demonstrating in vivo efficacy. With the 10 mg/kg dose the tumors shrunk permanently in all mice and did not grow again during the follow-up period of 100 days. With the 3 mg/kg dose the growth of the tumors was arrested to a stable disease in all mice. However, in the latter treatment group the tumor recurred in one mouse after 80 days (Figure 4b).

To initially study tolerated doses of MMAU ADCs in vivo, mice were given higher doses, 30 mg/kg, 50 mg/kg and 100 mg/kg, of DAR = 8 ADCs. In one mouse with HSC-2 xenograft tumor, four i.v. doses of 30 mg/kg anti-EGFR DAR = 8 MMAU ADC were given with one week intervals. The tumor size decreased, while no clinical signs of toxicity were observed during a follow-up period of one month after the last dosing and the weight of the mouse increased steadily from 22 to 28 g during the experiment. In another experiment, 50 mg/kg and 100 mg/kg single doses of DAR = 8 MMAU ADCs were given i.v. to non-tumor bearing mice, one mouse per dose group, and they were similarly followed. No signs of toxicity and no weight loss were observed during the follow-up period in either mouse.

Pharmacokinetics of trastuzumab-MMAU ADCs were studied in mice for both DAR = 4 and DAR = 8 ADCs (Figure 4c). Following a single 10 mg/kg i.v. dose, the serum concentration was followed for one week as a function of total human IgG concentration similarly as described previously to document the initial fast clearance following ADC administration [3]. The area under curve (AUC) of DAR = 8 MMAU ADC was decreased 31% compared to DAR = 4. The experiment was not continued to estimate the half-lives of the ADCs, since it has been established that due to instability of the maleimidocaproyl-peptide linker the apparent half-lives of ADCs with different initial DAR values approach each other during a longer experiment [3].

### 3.4. Efficacy in In Vitro 3D PDX Model

**Figure 5:**
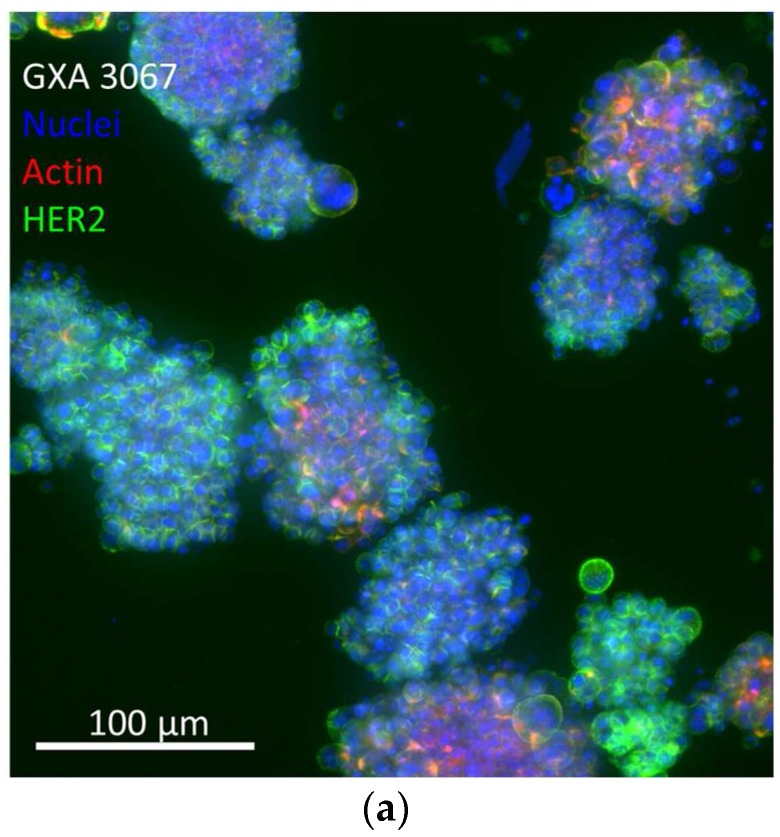

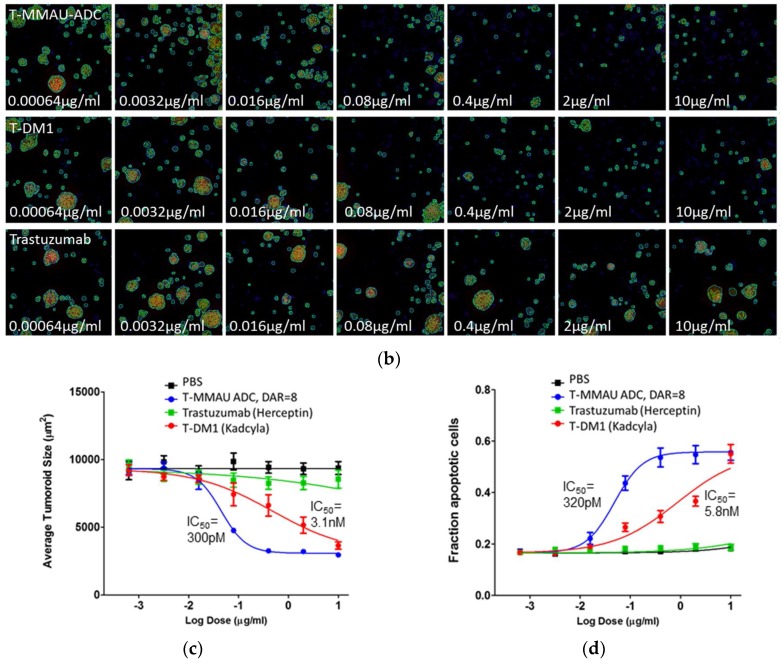
In vitro 3D patient-derived xenograft (PDX) model. (**a**) GXA3067 is a HER2 overexpressing gastric tumor PDX model; (**b**) Size-filtered tumoroids were seeded in 384-well plates and treated with increasing concentrations of ADCs, the trastuzumab-based DAR = 8 MMAU ADC (T-MMAU ADC, upper row) and T-DM1 (Kadcyla^®^, middle row); as well as the naked antibody trastuzumab (bottom row). (**c**) The DAR = 8 MMAU ADC was 10× more effective in reducing tumoroid size than T-DM1, while trastuzumab had only a moderate effect. (**d**) The DAR = 8 MMAU ADC was 18× more effective in inducing apoptosis than T-DM1, while trastuzumab did not have an effect. IC_50_ values are marked in the figure panels and they were determined using curve fitting by nonlinear regression as the concentration of the drug that causes 50% inhibition of cell viability compared to maximum inhibition. The experiments were made with eight replicates. Error bars show the standard error of the mean.

For a head-to-head comparison of the DAR = 8 MMAU ADC with a clinically approved ADC, Kadcyla^®^, we employed an in vitro 3D HER2 overexpressing gastric tumor PDX model GXA3067 (Figure 5) that more closely resembles the in vivo situation than monolayer culture [8]. In the 3D experiments, concentration series of trastuzumab (Herceptin^®^), T-DM1 (Kadcyla^®^) and trastuzumab-based DAR = 8 MMAU ADC were applied to the gastric cancer tumoroids (Figure 5b), and their effect on tumoroid size (Figure 5c; as a measure of cytotoxicity) and fraction of apoptotic cells (Figure 5d) were quantitated. Both ADCs inhibited tumoroid growth and induced apoptosis in the in vitro 3D PDX model. However, based on the IC_50_ values the DAR = 8 MMAU ADC was 10–18× more effective, respectively, than T-DM1 (Figure 5c,d). The anti-HER2 ADC T-DM1 comprises an anti-tubulin payload DM1 with an average DAR = 3.5 [12].

## 4. Discussion

Despite continuing advances in treating cancer, there are still a large number of patients who progress to advanced metastatic disease without a curative treatment option. Thus, there is a great unmet medical need and the effort is ongoing to develop novel therapeutic strategies such as ADCs. A handful of ADCs has already had remarkable clinical success [1], but challenges in their development remain. Many ADC payloads and linkers suffer from hydrophobicity and poor stability, limiting the available DAR to 2–4 [3,13,14]. In fact, many ADCs suffer from compromised biodistribution and pharmacokinetic profiles even with low drug loading. Furthermore, many technologies are limited to random conjugation of the drug to the antibody leading to heterogeneity of the ADCs [15]. These properties are among the causes for the narrow therapeutic window often seen with ADCs.

Efforts have been made to develop more hydrophilic auristatins, such as MMAF. However, MMAF suffers from loss of bystander kill activity that is considered important in targeting tumors with heterogeneous target antigen expression [16]. Indeed, after several years of work to generate improved auristatins, MMAE is still one of the most widely used payloads, also on the very recent ADCs to enter clinical development. In addition to its high cytotoxic activity towards both target and bystander cells [17], MMAE has relatively good tolerability in patients. Auristatins have yet another advantage, being tubulin inhibitors with an inherent property to selectively induce apoptosis in rapidly dividing cells such as cancer cells [18]. The major drawback of MMAE is its hydrophobicity, which leads to accelerated clearance of the ADC from circulation [3], followed by liberation of free payload in off-target locations.

In the present report, we have described a novel auristatin glycoside antimitotic agent, MMAU (Figure 1a). MMAU as a free payload has much lower cytotoxic activity (Figure 3c) than MMAE that has IC_50_ value in the low-nM range [10,19], presumably owing to the bulky, hydrophilic and charged C-terminal glycoside group that impairs passive transport through the cellular membrane. In contrast, we demonstrated that the activities of MMAU are potentiated several orders of magnitude when the drug is attached through a peptide linker to an internalizing antibody that is taken up by target cells. While having very low toxicity if the toxin is prematurely released, the DAR = 8 MMAU ADC had low-picomolar potency to the target cells (Figure 3a–c), and showed efficient kill of even cell lines expressing low copy numbers of the targeted tumor antigen (Figure 3b). Further, as the payload is converted to MMAE through cellular metabolism, the MMAU has a favorable bystander kill profile (Figure 3c) that is lacking from hydrophilic payloads such as MMAF.

The striking benefit of MMAU is its highly hydrophilic nature allowing for conjugation to DAR = 8 without tendency to ADC aggregation (Figure 2e,f) and with apparent hydrophobicity similar to DAR = 3–4 MMAE ADCs as analyzed with HIC (Figure 2g,h). In our view, the generated novel auristatin glycoside combines the best of two worlds: (1) a highly hydrophilic payload allowing for high-DAR applications with low toxicity as a free drug and (2) high cytotoxic activity towards target cells together with efficient bystander kill after internalization and metabolic processing of MMAU to MMAE.

The data from 3D in vitro PDX assay clearly shows the apoptosis-inducing activity of MMAU in concordance with other auristatin payloads [18], thus demonstrating the anticipated mode of action. In this model, the DAR = 8 MMAU ADC outperformed Kadcyla^®^ both in cancer cell growth inhibition and apoptosis induction.

In vivo, we found that the DAR = 8 MMAU ADC was well tolerated at the utilized doses in mice. In vivo activity was demonstrated using an anti-EGFR ADC and EGFR-expressing target cell line. It should be noted that this model is particularly challenging to MMAU which requires true lysosomal targeting for processing and activation. EGFR is known to circulate mainly to endosomes and back to the cell surface whilst only a fraction of the receptor reaches lysosomal compartment. The significance of this finding may become evident though further toxicological analyses, in which MMAE- and MMAU-based ADCs should be quite distinct if systemically released drug contributes to ADC toxicity. We are in the process of assessing several aspects of MMAU payloads and ADCs, including more detailed analysis of the toxicity profile and assessing of the therapeutic index. In preliminary in vivo studies we have thus far dosed DAR = 8 MMAU ADCs up to 100 mg/kg in mice without any signs of toxicity, although the maximum tolerated doses of both the free MMAU payload and MMAU ADCs remain as subjects of further study. Corresponding DAR = 8 MMAE ADCs have maximum tolerated dose of 50 mg/kg in mice [3]. However, tolerability of DAR = 8 MMAU ADC in comparison to DAR = 8 MMAE ADC was not directly compared and remains an open question.

Our current work is focusing on using improved, more lysosome specific linkers to overcome the known shortcomings of the MC-Val-Cit-PABC linker system including the retro-Michael exchange, susceptibility to extracellular release by proteases, and in mouse models cleavage of the peptide bond by mouse carboxyesterase-1 [20]. MC-Val-Cit-PABC linker is also known to increase the relative hydrophobicity of the drug-linker compound [4] thus partly compromising the beneficial hydrophilicity of MMAU. In the present initial pharmacokinetics study in mice, DAR = 8 trastuzumab-MMAU ADC had 31% lower AUC than the corresponding DAR = 4 ADC. We did not perform a direct comparison with DAR = 8 MMAE ADC, although it has been reported for other antibody-based ADCs that DAR = 8 MMAE ADC has clearly lower AUC than DAR = 4 MMAE ADC [3]. The present results encourage combining MMAU with more hydrophilic linkers to further improve the hydrophilicity of the whole ADC molecule. Such work is ongoing and will be reported elsewhere.

Human cells universally express β-glucuronidase across different tissues [21], even so that the *GUSB* gene encoding the enzyme is commonly used as a housekeeping gene marker in studies of gene expression levels. Moreover, β-glucuronidase enzyme shows increased activity in cancer tissues and studies have reported using glucuronide glycosides as activating groups in cancer-targeting prodrugs [22]. Within the ADC field, glucuronides have been successfully utilized in duocarmycin payloads [23] as well as in glucuronidase-cleavable linkers [24]. Thus the available evidence supports a conclusion that the β-glucuronide glycoside of MMAU will be effectively cleaved inside target cells after ADC internalization. However, there exists an extremely rare inherited lysosomal storage disease caused by β-glucuronidase deficiency called Sly disease or mucopolysaccharidosis type VII, which has a prevalence of about 1/1,000,000 [25]. People with such disease should be less responsive to treatment with an MMAU ADC due to decreased ability to liberate MMAE in its active form from the MMAU payload.

In conclusion, the present findings indicate that MMAU is a promising novel payload to overcome many challenges of ADC technology. Increased hydrophilicity of the payload contributes to the ADC’s hydrophilicity, stability and safety to non-target cells, while significantly improving cytotoxicity by increasing the allowed DAR and enabling bystander efficacy.

## Supplementary Materials

The following are available online at http://www.mdpi.com/2073-4468/7/2/15/s1, Figure S1: MALDI-TOF mass spectrum of MC-VC-PAB-MMAU (see insert): [M+Na]^+^ m/z1505.04 (calculated 1514.80), [M-H+2Na]^+^ m/z 1537.03 (calculated 1536.78).
