# Supplementary material for: Hydrophilic Auristatin Glycoside Payload Enables Improved Antibody-Drug Conjugate Efficacy and Biocompatibility
Source: Antibodies (Basel). 2018 Mar 22;7(2):15. doi: 10.3390/antib7020015 (PMC6698876; doi:10.3390/antib7020015)

Supplementary Figure S1. MALDI-TOF mass spectrum of MC-VC-PAB-MMAU (see insert): $[M+Na]^+$ m/z 1505.04 (calculated 1514.80), $[M-H+2Na]^+$ m/z 1537.03 (calculated 1536.78).

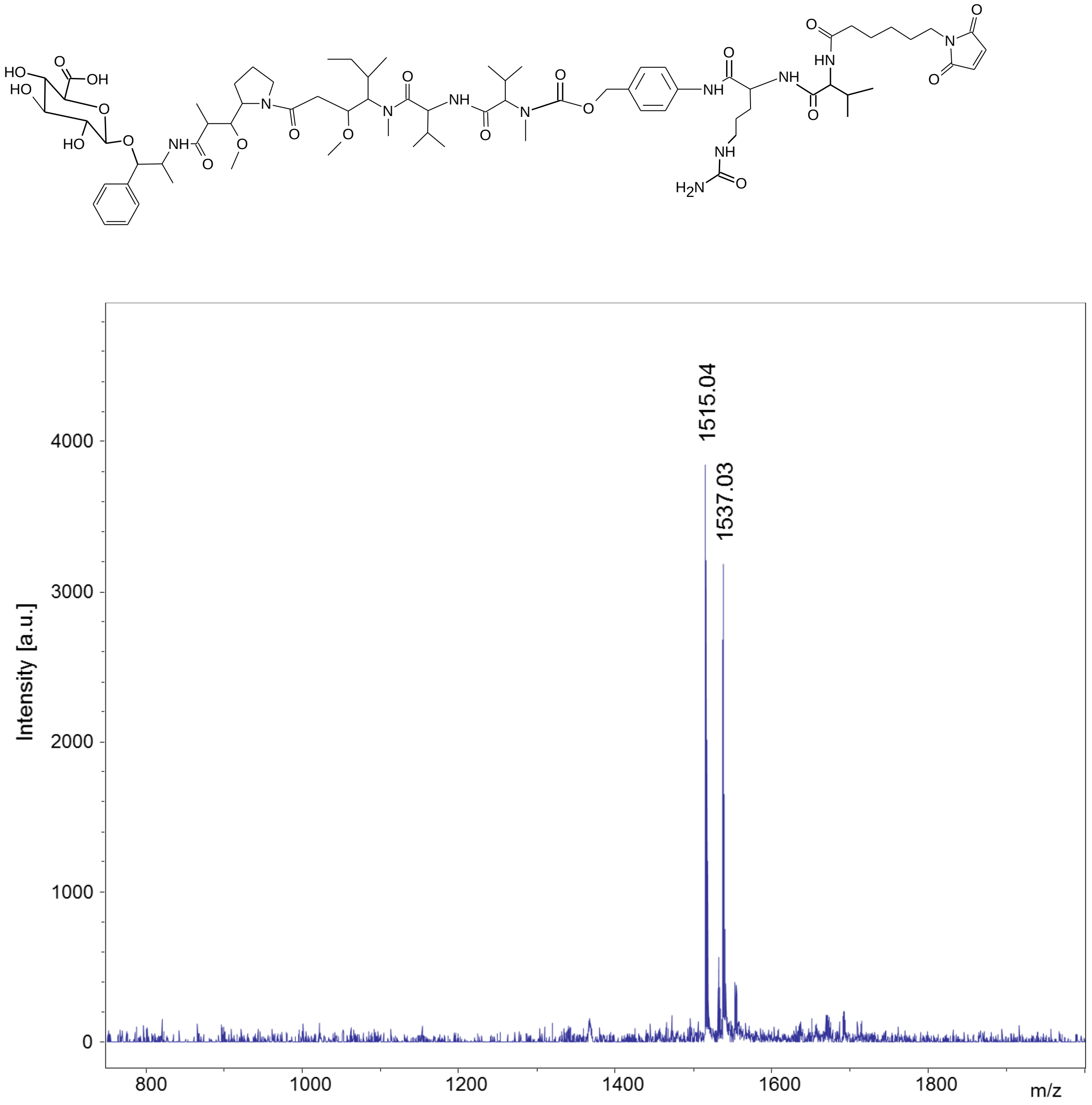

Supplement: Supplementary file 1 [file antibodies-07-00015-s001.pdf]
